# Hydrogel of Thyme-Oil-PLGA Nanoparticles Designed for Skin Inflammation Treatment

**DOI:** 10.3390/gels10020149

**Published:** 2024-02-18

**Authors:** Camila Folle, Natalia Díaz-Garrido, Mireia Mallandrich, Joaquim Suñer-Carbó, Elena Sánchez-López, Lyda Halbaut, Ana M. Marqués, Marta Espina, Josefa Badia, Laura Baldoma, Ana Cristina Calpena, Maria Luisa García

**Affiliations:** 1 Department of Pharmacy, Pharmaceutical Technology and Physical Chemistry, Faculty of Pharmacy and Food Sciences, University of Barcelona, 08028 Barcelona, Spain; camilafolle@ub.edu (C.F.); mireia.mallandrich@ub.edu (M.M.); jsuner@ub.edu (J.S.-C.); m.espina@ub.edu (M.E.); anacalpena@ub.edu (A.C.C.); marisagarcia@ub.edu (M.L.G.); 2 Department of Biochemistry and Physiology, Biochemistry and Biomolecular Science, University of Barcelona, 08028 Barcelona, Spainjosefabadia@ub.edu (J.B.); lbaldoma@ub.edu (L.B.); 3 Institute of Biomedicine, University of Barcelona, 08028 Barcelona, Spain; 4 Research Institute Sant Joan De Déu (IR-SJD), 08950 Barcelona, Spain; 5 Institute of Nanoscience and Nanotechnology (IN2UB), University of Barcelona, 08028 Barcelona, Spain; 6 Department of Biology, Healthcare and Environment, Faculty of Pharmacy and Food Sciences, University of Barcelona, 08028 Barcelona, Spain; ammarques@ub.edu

**Keywords:** thyme oil, polymeric NPs, antimicrobial, anti-inflammatory, antioxidant, wound healing

## Abstract

Thyme oil (THO) possesses excellent antibacterial and antioxidant properties which are suitable for skin inflammatory disorders such as acne vulgaris. However, THO is insoluble in water and its components are highly volatile. Therefore, these drawbacks may be overcome by its encapsulation in biodegradable PLGA nanoparticles (THO-NPs) that had been functionalized using several strategies. Moreover, cell viability was studied in HaCat cells, confirming their safety. In order to assess therapeutic efficacy against acne, bacterial reduction capacity and antioxidant properties were assessed. Moreover, the anti-inflammatory and wound-healing abilities of THO-NPs were also confirmed. Additionally, ex vivo antioxidant assessment was carried out using pig skin, demonstrating the suitable antioxidant properties of THO-NPs. Moreover, THO and THO-NPs were dispersed in a gelling system, and stability, rheological properties, and extensibility were assessed. Finally, the biomechanical properties of THO-hydrogel and THO-NP-hydrogel were studied in human volunteers, confirming the suitable activity for the treatment of acne. As a conclusion, THO has been encapsulated into PLGA NPs, and in vitro, ex vivo, and in vivo assessments had been carried out, demonstrating excellent properties for the treatment of inflammatory skin disorders.

## 1. Introduction

Skin inflammatory disorders include several pathologies, and, among them, one of the most prevalent is Acne vulgaris (acne). Acne affects 80% of adolescents, and this condition often persists until early adulthood and may cause skin scarring [1]. This pathology is concurrent with high sebum production, the follicular keratinization of the pilosebaceous ducts, and a dysregulation of the skin microbiome [2]. Regarding the latter, *Cutibacterium acnes* (*C. acnes*) constitutes the major commensal of the normal skin flora, but it has been observed that this also contributes to acne pathogenesis when found in sebum-rich areas [2]. In addition, stress oxidative biomarkers have also been found in acne [3].

Despite these facts, current treatments for acne consist mainly of antibiotics and isotretinoin [2], which have shown limited efficacy and often contribute to dysregulation of the skin microbiome. Therefore, novel therapies aimed at balancing the skin microbiome via acting on the inflammation, wound-healing, and oxidative stress involved in acne, are of great relevance.

Recent studies have shown that essential oils and plant herbs may possess suitable properties for acne treatment. In fact, most clinical trials showed that herbal medicines significantly reduced acne lesions (both inflammatory and non-inflammatory), and were able to decrease acne severity (in mild–moderate acne) [4]. Among several natural compounds, thyme oil (THO) possesses excellent pharmaceutical properties [5,6]. THO is a product of the steam distillation of the fresh flowering aerial parts of *Thymus vulgaris* L. and/or *Thymus zygis* L. s, and it contains 37–55% thymol and 0.5–5.5% carvacrol [7]. Moreover, THO demonstrated an antibacterial effect on Gram-positive and Gram-negative bacteria, and has antiviral, antifungal, antioxidant, anti-inflammatory, and spasmolytic activities [7]. Specifically, against bacteria, both thymol and carvacrol act in a similar manner, based on being integrated on the lipid layer of the pathogen and increasing the surface curvature, causing changes in the membrane structure and the destabilization of the lipid layer, as well as decreases in elasticity, fluidity increases, and the modifications of membrane proteins [7]. However, THO is insoluble in water, and its components are highly volatile, therefore, it could lead to degradation if exposed to extensive light, oxygen, or extremely high temperatures [8].

To overcome these drawbacks, THO can be encapsulated into biodegradable nanoparticles (NPs). These NPs will enhance THO skin penetration and provide a controlled release [9,10]. Among several polymers, poly-(D,L)-(lactic-co-glycolic acid) (PLGA) has previously expressed excellent properties for pharmaceutical and skin application [10,11]. PLGA is biocompatible and biodegradable, as well as being approved by the Food and Drug Administration (FDA) and the European Medicines Agency (EMA).

However, nanoparticle aqueous suspensions may be challenging to apply at a clinical level. Therefore, in order to enhance their skin adhesion, NPs may be dispersed into semi-solid systems, such as hydrogels, which can serve as suitable vehicles for the administration of drugs or nanoparticulate systems to the skin, offering additional occlusive properties [12].

Taking this into account, in this study, THO has been encapsulated into PLGA NPs (THO-NPs), and this formulation has been surface-functionalized using different strategies, and their physicochemical properties had been studied. Moreover, NPs and free THO had been dispersed in two different gelling systems, and their rheological properties had been examined. Additionally, the in vitro antimicrobial, antioxidant, anti-inflammatory, and wound-healing capacity of the developed formulations had been evaluated. After these assessments, the ex vivo skin antioxidant capacity, as well as the in vivo skin biomechanical properties have been investigated.

## 2. Results and Discussion

### 2.1. Characterization and Optimization of THO-NPs

THO-NPs were prepared using the solvent displacement method, and their surface was functionalized using several molecules, such as Phosphatidylcholine (PL) or Poloxamer 188 (PX) when negatively charged, and chitosan (CS) when positively charged, referred to as THO-NP-L-, THO-NP-P-, and THO-NP-P-C+, respectively. The morphometry of the developed formulations, determined by dynamic light scattering (DLS), and their physicochemical stability, stored at 4 °C during 6 months, are shown in Table 1. All formulations presented suitable average size (Z_av_) and polydispersity index (PI) values for skin administration (between 100 and 400 nm) [13,14,15]. The lowest increase in particle size during storage can be observed for the particle containing PX, whereas the highest increase is observed in particles containing CS. In the case of zeta potential (ZP), the values tend to decrease during storage, therefore, the best stability could be expected for the highest value; in this case, those containing PL. Moreover, the long-term physicochemical stability of the surface-functionalized THO-NPs was observed 6 months, maintaining the skin-optimal parameters (Table 1), as well as the appearance and organoleptic properties.

### 2.2. In Vitro Bacterial Viability Reduction

**Figure 1:**
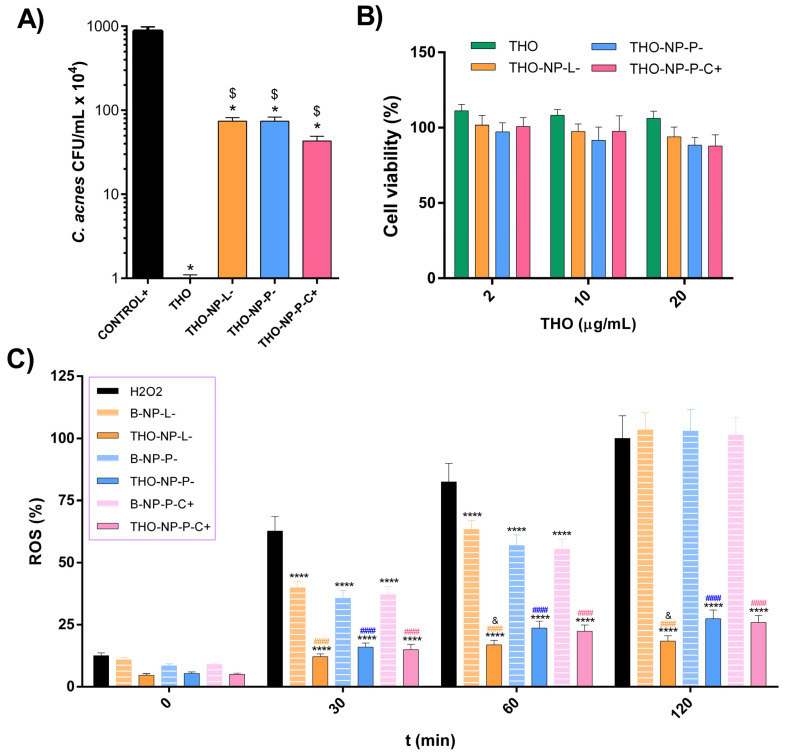
Viable count experiments of cells treated with THO and THO-NPs. (**A**) Antibacterial capacity of the formulations against *C. acnes*. Values are expressed as mean ± SD (*n* = 3), and are represented as (* *p* < 0.0001): against control (*C. acnes* without treatment) and (^$^ *p* < 0.0001): against THO. (**B**) Cell viability of HaCaT cells (24 h incubation) at different concentrations. Values represent the Mean ± SEM (*n* = 3). (**C**) Antioxidant activity in HaCaT cells H_2_DCFDA labelled and challenged with H_2_O_2_. Data are expressed as the Mean ± SD of quantified ROS (%), *n* = 8, (**** *p* < 0.0001 versus control; ^####^ *p* < 0.0001 versus corresponding blank NPs, ^&^ *p* < 0.05 vs. other THO-NPs). Statistical analysis developed either by *t*-test or by one-way ANOVA (Tukey’s Multiple Comparison Test).

The in vitro antimicrobial activity of THO and THO-NPs when attempting to eradicate *C. acnes* was evaluated by the suspension test within 30 minutes of contact. All samples presented statistically significant reductions of bacterial viability when compared to the control. Results for the developed surface-functionalized THO-NPs presented no statistically significant differences between them. However, THO showed a fast and complete microbial growth depletion within this short period (Figure 1A).

### 2.3. Cytotoxicity in HaCaT Cells

The cell viability of HaCat cells in contact with surface-functionalized THO-NPs for 24 h (2, 10 and 20 µg/mL) was measured using the MTT (3-(4,5-Dimethylthiazol-2-yl)-2,5-diphenyl tetrazolium bromide) method. Results showed that all formulations tested presented cell viabilities above 80% (Figure 1B).

### 2.4. Antioxidant Activity

The antioxidant activity of THO-NPs was evaluated using reactive oxygen species (ROS) quantification in H_2_DCFDA-labelled HaCaT cells, stressed with H_2_O_2_ [16]. Treatment with surface-functionalized THO-NPs significantly reduced intracellular ROS, presenting significant differences against the control (*p* < 0.0001) and against their corresponding blank NPs (*p* < 0.0001) (Figure 1C). As can be observed, the unloaded surface-functionalized PLGA-NPs (blank NPs) have shown a mild activity against ROS along the reaction. However, after 2 h, no effect was expressed, since the cells reached 100% ROS. On the other hand, THO-NPs presented a strong antioxidant activity, as they could scavenge H_2_O_2_ free radicals, maintaining low levels of ROS at the cellular level. Additionally, THO-NP-L- presented significant statistical differences when compared to THO-NP-P- at 60 and 120 min, with it being the formulation with the higher antioxidant capacity. In contrast, unloaded NP-L- presented the lowest activity when compared to the other blank NPs. Therefore, it can be observed that a synergic effect of THO and PL boosts the antioxidant potential of the NPs.

### 2.5. Anti-Inflammatory Activity

**Figure 2:**
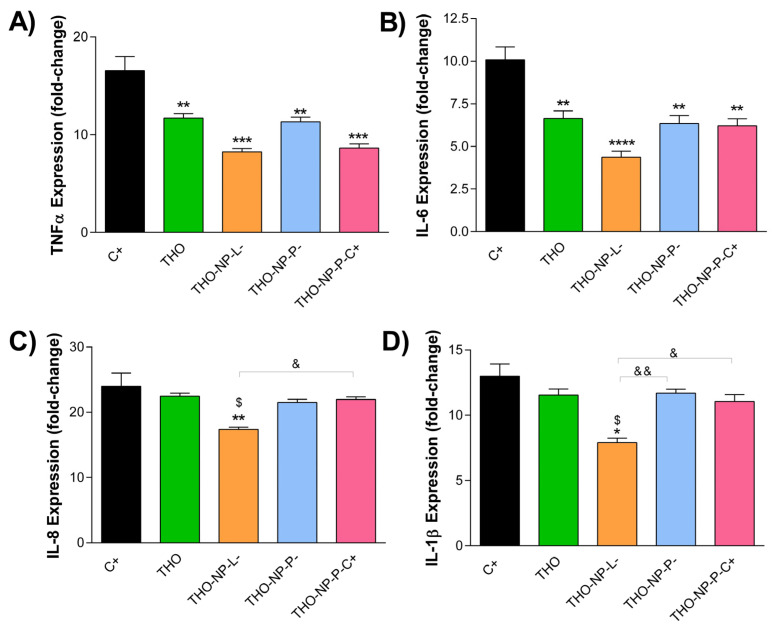
Gene expression values of inflammatory cytokines in HaCaT cells infected with *C. acnes* and treated with THO or THO-NPs. Relative mRNA levels of (**A**) TNF-α, (**B**) IL-6, (**C**) IL-8, and (**D**) IL-1β, measured by RT-qPCR, with β-actin as the endogenous control. Values (Mean ± SEM, *n* = 3) are expressed as fold-change compared to untreated HaCaT cells (control+). Statistical analysis was performed by one-way ANOVA, followed by Tukey’s Multiple Comparison Test, and is represented as: * *p* < 0.05, ** *p* < 0.01, *** *p* < 0.001 or **** *p* < 0.0001: versus positive control (C+), ^$^ *p* < 0.05: versus THO, and ^&^ *p* < 0.05 or ^&&^ *p* < 0.01: versus other THO-NPs.

The anti-inflammatory capacity of THO and THO-NPs against *C. acnes* was studied by adding directly to the HaCaT cells a freshly prepared inoculum to induce inflammation. Gene expression encoding the inflammatory cytokines were analyzed after *C. acnes* infection in the cells pre-treated with THO or THO-NPs. Cells challenged only with *C. acnes* were used as a positive control. Results illustrated in Figure 2 show that THO-NP-L- presented higher activity for all gene tested, compared to the other formulations, presenting statistically significant differences, also against free-THO. For TNF-α (Figure 2A), and THO-NP-P-C+ presented higher activity, although THO and THO-NP-P- were also effective. In the case of IL-1β (Figure 2B), all formulations showed significant activity, however, only THO-NP-L- presented outstanding efficacy. For IL-6 (Figure 2C) and IL-8 (Figure 2D), no relevant activity was observed, despite THO-NP-L- showing statistically significant differences compared to the control, THO, and other THO-NPs. Other authors confirmed that the anti-inflammatory activity of THO, depending on the plant species used, is variable, except for those that are dose dependent [17]. Therefore, the formulation with surface functionalization and PL has the highest potential to be used to treat acne skin inflammation. THO-NP-L- presented the highest activity obtained in all cases, which confirms the hypothesis that PL acted as a good booster of THO-NP activity.

### 2.6. Wound Healing

**Figure 3:**
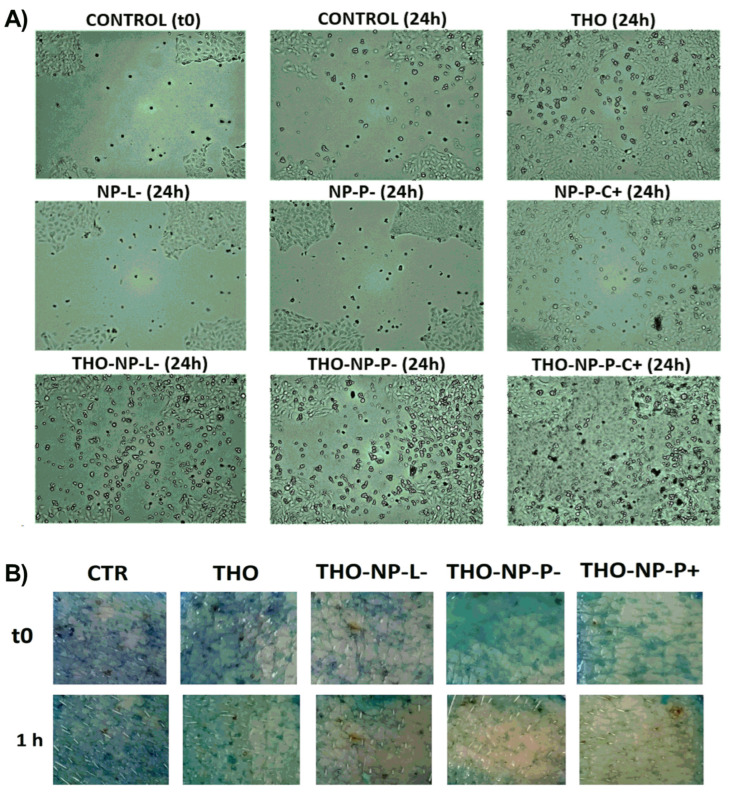
Skin cell regeneration studies: (**A**) In vitro wound healing activity in HaCaT cells scratch treatment. Images recorded prior to incubation (Control t0) and after 24 h, untreated (control 24 h) and treated, with THO, THO-NP-L-, THO-NP-P-, THO-NP-P-C+, and their corresponding blank NPs (NP-L-, NP-P-, NP-P-C+). (**B**) Ex vivo antioxidant activity of THO, THO-NP-L-, THO-NP-P-, THO-NP-P-C+, or water (as the control), as shown by the methylene blue reduction on the skin surface.

In this study, the scratch assay was performed to evaluate the wound-healing treatment activity in HaCaT cell lines through observing cell proliferation. Results shown in Figure 3A indicate that THO-NPs showed higher wound-healing capacity than the control, THO, and their corresponding blank NPs. Additionally, surface composition seemed to influence these parameters, since enhanced cell proliferation and wound closure was observed, especially in the case of THO-NP-P-C+, which could be related to the positively charge, or to the synergic activity of the formulation compounds (THO, PX, and CS). These results are similar to the ones previously found by our group, where NP-P-C+ (loading-thymol) also presented outstanding wound-healing efficacy in HaCaT cells [10]. The previously proven cell viability and boosted antioxidant activity of THO-NP-L would probably increase the wound-healing activity for skin application in a long-term treatment. In fact, all samples presented outstanding results in wound closure, and would thus be suitable and efficient for healing skin inflammation and acne scars.

### 2.7. Ex Vivo Skin Antioxidant Efficacy

THO and surface-functionalized THO-NP antioxidant activity were assessed using ex vivo pig skin incubated for 1 h (Figure 3B). The results showed that all tested formulations showed antioxidant activity, as observed by higher color reductions compared to the control. A slightly higher activity can be observed for THO-NPs when compared qualitatively to THO. Thus, it can be predicted that the encapsulated form and/or the synergic effect with the surface-functionalized compounds would improve the activity on the skin.

### 2.8. Characterization and Stability of the Hydrogels

**Figure 4:**
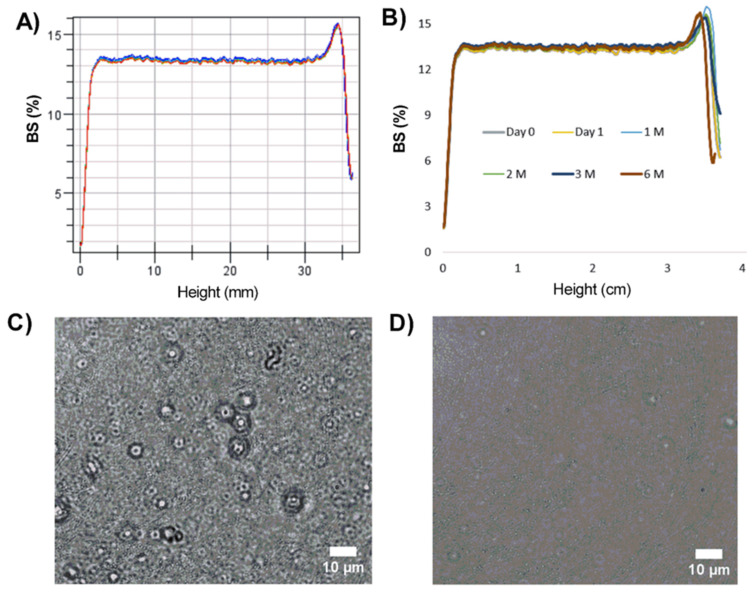
The long-term stability of GC-THO-NP-L- by means of the backscattering profile, measured by Turbiscan^®^ Lab Expert (**A**) for 24 h (1 scan every h, represented by the blue and red lines) and (**B**) under storage at RT for 6 months (1 scan every selected measurement day). Optical micrographs (X63) of (**C**) GC-THO-NP-L- and (**D**) GC-THO (scale bars representing 10 µm).

The incorporation of the optimized NPs into a carbomer-based hydrogel (GC) was successfully conducted (GC-THO-NP-L-), and no relevant variation on the particle size could be observed (Table 2) after 6 months, maintaining the optimal skin parameters. In the case of PI, the values have increased two-fold since the overall value also measures the macrogel matrix. The ZP of the hydrogel was not measured since the value would not correspond to the NP surface, but instead to the overall system charge of the polymeric matrix of the macrogel. The appearance and organoleptic properties were also evaluated for the developed gels, including one containing free THO; both maintained their initial properties, remaining stable. However, both formulations were obtained with a slightly different appearance: GC-THO relies on a semi-solid soft gel and GC-THO-NP-L- a serum-like gel. The stability of hydrogels was assessed using the Turbiscan^®^ Lab Expert (Iesmat, Madrid, Spain). The backscattering signal (BS) of GC-THO-NP-L- was measured to predict the short-term future stability behavior; the data are shown in Figure 4A. Thus, it can be observed that the system is stable and absent of sedimentation, creaming, or flocculation. The stability was also maintained during storage, as seen in Figure 4B, where no relevant variation of the BS% can be observed for 6 months at 4 °C. Additionally, the microbial sterility tested for the aqueous surface-functionalized THO-NPs and for both the developed hydrogels was confirmed after 6 months by the absence of microbial growth in the culture plates. Therefore, the developed formulations, aqueous solutions, and hydrogels provided good physicochemical, organoleptic, and sterility stability during storage.

### 2.9. Rheological Studies

**Figure 5:**
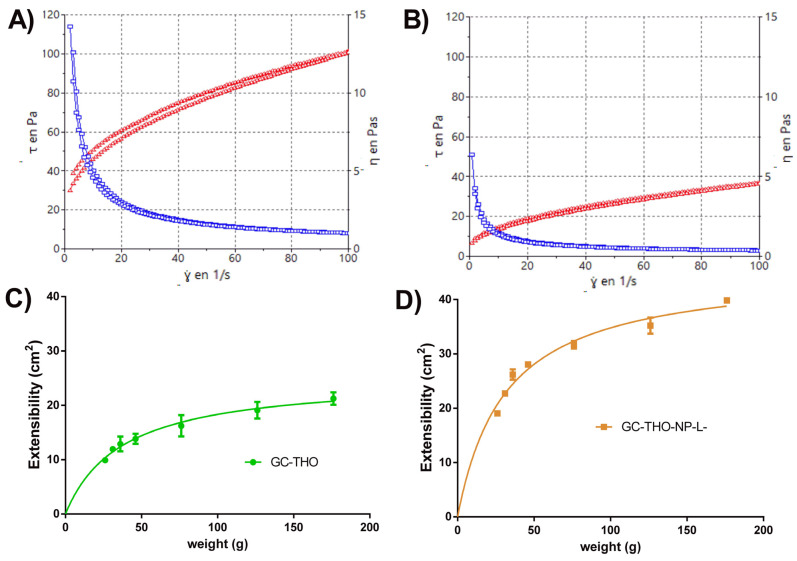
The characterization of the developed hydrogels, GC-THO and GC-THO-NP-L- (left and right, respectively). (**A**,**B**) Rheological profile with viscosity curve (blue line) and flow curve (red line) (*τ*: shear stress, *ץ:* shear rate, *η*: apparent viscosity). (**C**,**D**) Extensibility profile as the function of increasing weights, adjusted to the Hyperbola equation.

The rheological behavior of the developed hydrogels (GC-THO and GC-THO-NP-L-) was determined, highlighting that the rheological results were dependent on the shear rate (Figure 5A). Both formulations exhibited non-Newtonian shear-thinning behavior, with a consistent decrease in viscosity and an increasing shear rate between 1 and 100 s^−1^. The mathematical model that best fitted the experimental data was the Herschel–Bulkley equation. The viscosity was determined at a constant rate 100 s^−1^, being 1.01 ± 0.00 and 0.37 ± 0.00 (Pa·s) for GC-THO and GC-THO-NP-L-, respectively (Table 3). Thus, it can be observed that the viscosity obtained for the hydrogel incorporating the PLGA-NP-loaded THO was lower than the hydrogel containing free-THO. This can be explained via the fact that the aqueous NPs, when formed, present low pH values, and, therefore, since carbomer is a pH-dependent gelling agent, by adjusting the same pH value for both formulations, different viscosity values emerge.

### 2.10. Extensibility Studies

The extensibility profiles of GC-THO and GC-THO-NP-L- were adjusted to the Hyperbola equation and then represented as the function of the increasing applied weight (Figure 5B). The parameters obtained from this mathematical model are shown in Table 3. The maximum extensibility values (*E_max_*) were found to be 25.09 cm^2^ and 45.71 cm^2^, respectively, being 1.8 times higher for the hydrogel containing the NPs. The extensibility constants (*K_m_*) were 37.02 g and 31.44 g, respectively, being the necessary weight applied to reach half of the maximum extensibility. Comparing both hydrogels, the *K_m_* values were similar, but the extensibility capacity of GC-THO-NP-L- was much higher, which is more convenient for skin inflammatory pathologies. Moreover, by comparing these results with the ones obtained above for the rheology, the extensibility of the gel was found to be inversely proportional to the viscosity value, which is also in agreement with the type of topical formulation (appearance) obtained.

### 2.11. In Vivo Skin Biomechanical Properties

**Figure 6:**
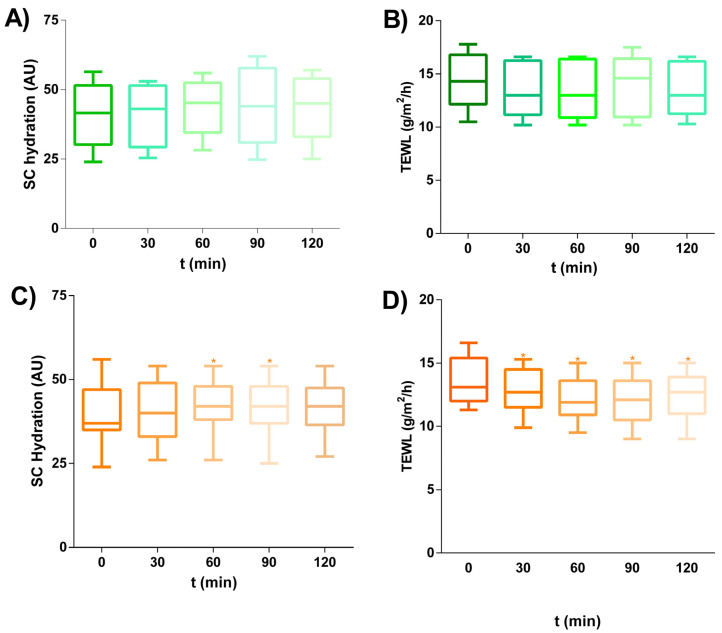
In vivo skin biomechanical properties of GC-TH and GC-TH-NLC-L- (up and down, respectively), measured on the forearm of healthy volunteers for SC skin hydration (Corneometer^®^, **A**,**B**) and skin barrier functions (TEWL^®^, **C**,**D**) (*n* = 12). Statistical analysis was performed using ANOVA non-parametric Wilcoxon paired tests, comparing each measure against the basal value (t0), * indicates *p* < 0.05 against the initial value.

The biomechanical properties of GC-THO and GC-THO-NP-L- were evaluated on the forearms of 12 voluntaries, measuring the basal levels both before and after the application of the stratum corneum (SC) hydration (Corneometer^®^) (Figure 6A,B) and the transepidermal water loss (TEWL^®^) (Figure 6C,D), respectively. The basal levels where selected based on the values that were found acceptable for moderately compromised skin barriers (10–20 g/m^2^/h, TEWL) and a variety of SC hydration values (hydrated > −45, dry < 45 and very dry < 30) [18,19]. The results provided no statistically significant differences in the case of GC-THO. Moreover, GC-THO-NP-L- showed a significant decrease in TEWL (*p* < 0.05), while GC-THO provided no effect, which could possibly be associated with the essential oil properties of the skin. Previous authors stated that essential oils may act as a skin penetration booster by disrupting the intercellular skin lipids and thus changing the cellular membrane fluidity [20]. This can, therefore, raise the hypothesis that this effect on the lipids of the skin barrier and the cell-regenerating properties of THO can be balanced for the skin application, especially with the excipient also containing humectant ingredients. In the case of the PL on the surface of THO-NP-L-, this compound would likely boost the activity, and improve the skin barrier function within a long-term skin application treatment.

From a different perspective, GC-THO-NP-L- showed a statistically significant decrease in TEWL and an increase in SC hydration. In this sense, THO-loaded NP surfaces functionalized with PL would likely improve the skin barrier function, due to the small particle size, which could fill the SC gaps via restoring the skin lipids using the biomimetic PL. However, a long-term skin application treatment would be required in order to explore the greater effects of these formulations.

### 2.12. Discussion

Acne constitutes the eighth most common skin disease, affecting 9% of the population worldwide, and approximately 85% of individuals aged 12–24 years [21]. Therefore, the development of novel therapies is crucial, as this could greatly increase the quality of life of these patients. For this reason, in this study, THO has been encapsulated into PLGA NPs. Moreover, THO NPs have been functionalized using PL, PX, or CS, and they have been physiochemically characterized. In this sense, the most suitable physicochemical properties were obtained using THO-NP-L- presenting a high surface charge, which may contribute to long-term stability. Moreover, the structure of PL is similar to the skin, which would be appropriate for the skin-delivery treatment [22]. Using all the functionalized PLGA NPs, the antimicrobial activity was assessed, obtaining a strong response against *C. acnes*, especially in the case of THO. Comparing this result with our previously obtained results for thymol (the major component of THO) in an earlier work [10], this rapid antimicrobial activity of THO may be related to its synergistic activity, combined with the other constituents of this essential oil. Previous authors stated that essential oils possess strong antimicrobial or bactericidal activity due to their composition containing several aromatic structures [23]. Other studies claim that the essential oils first target as an antimicrobial substance is the bacteria cell wall and membrane, which causes increased membrane permeability, leading to cell leakage of proteins, genetic material, and membrane potential [24]. However, the complete eradication of *C. Acnes* may contribute towards an imbalance of the skin microbiome [2].

In addition, the safety of the functionalized NPs has been demonstrated in HaCaT cells, as values above 80% of the cell viability were obtained. These results were expected, since other authors have previously claimed safety of PLGA-based nanocarriers [9,10]. Using this cell line, the antioxidant capacity of the developed formulations was also elucidated at several time points. It can be observed that THO-NP-L- showed the best antioxidant properties, probably due to the synergistic effects of THO and PL, thus increasing the antioxidant potential of the NPs. Previous authors have stated that plant oil components (PL, phenolic aromatic, and antioxidants) are able to exhibit synergistic activity by providing antimicrobial, antioxidant, and anti-inflammatory properties, as well as promoting homeostasis of the skin barrier and wound healing [25]. These results are consistent with the anti-inflammatory activity, thus confirming that THO-NP-L- showed the most suitable results by being able to decrease all the inflammatory markers studied. Previous authors have also found improved antioxidant and anti-inflammatory results when using nanosystems with phosphatidylcholine [26].

Moreover, concerning the wound-healing activity, all surface-functionalized NPs showed suitable wound-healing activities. However, the most potent was obtained by THO-NP-P-C+, probably due to the chitosan positive charge, as well as its intrinsic pharmacological properties [27]. In this regard, the enhanced wound-healing activity combined with THO and chitosan has been previously reported, as a higher cellular proliferation was observed [6]. Moreover, THO has been advocated to play a role in leukocyte influx and also to decrease inflammatory compounds [28]. Antioxidant capacity was also studied ex vivo, showing that functionalized NPs obtained higher antioxidant properties than non-encapsulated THO. These findings are similar to those found in our earlier work with PLGA-loaded thymol NPs, presenting increased levels of activity when compared to the free dosage form [10].

Finally, THO-NP-L- were dispersed in a carbomer hydrogel (GC-THO-NP-L-), along with THO (GC-THO). Both demonstrated suitable stability for 6 months at 4 °C. In order to characterize the hydrogels, the rheological properties of GC-THO-NP-L- and GC-THO were studied. The best fit was obtained using the Herschel–Bulkley equation, which provides a general model for shear-thinning materials with pseudoplastic flow, which is ideal for skin applications. In addition, the extensibility of GC-THO-NP-L- was greater than that of GC-THO, which may improve skin application at a clinical level. Finally, THO-NP-L- and GC-THO were applied to human volunteers. The biomechanical properties of the skin showed that the GC-THO hydrogel did not alter skin properties, nor did it disrupt or hydrate the SC. In contrast, GC-THO-NP-L- provided TEWL reduction and slightly increased the SC hydration in a statistically significant manner, when compared to basal levels. Therefore, it can be concluded that the combined formulation ingredients (encapsulated THO, surface functionalization with phospholipids and humectants) were able to improve the skin barrier conditions. Moreover, PLGA is a biodegradable polymer, which will degrade into its monomers (lactic acid and glycolic acid); therefore, these compounds may help to modulate the skin pH, maintaining its acidity, which may be favorable for rebalancing the skin microbiome and treating acne inflammation.

## 3. Conclusions

In this work, THO-loaded PLGA NPs, including several surface modifications, were successfully obtained and physiochemically characterized. Functionalized nanosystems remained stable at 4 °C for up to 6 months. Moreover, in vitro cellular experiments confirmed their safety, since the cell viability was above 80% and THO-NPs provided significant antibacterial reduction. THO-NPs were antioxidant, both in vitro and ex vivo, and provided anti-inflammatory properties, the best anti-inflammatory performance being with THO-NP-L-. Moreover, THO-NPs also demonstrated wound-healing properties, which were enhanced in the case of chitosan-functionalized NPs.

Additionally, THO-NP-L and THO were dispersed in a carbomer hydrogel that was physiochemically characterized, demonstrating suitable stability, rheological properties, and extensibility. Moreover, GC-THO did not alter the biomechanical properties of the skin, whereas GC-THO-NP-L significantly increased the hydration of SC, and reduced trans-epithelial water loss significantly.

In conclusion, THO-NPs constitute suitable candidates for the treatment of skin inflammatory pathologies due to their suitable antibacterial, antioxidant, and anti-inflammatory properties.

## 4. Materials and Methods

### 4.1. Materials

PLGA Resomer^®^ RG 504H (lactide:glycolide 50:50, carboxylic terminal group, molecular weight 38,000–54,000 Da) was acquired from Boehringer Ingelheim (Ingelheim, Germany). Poloxamer 188 (PX), glycerin (GLY), and propylene glycol (PG) were supplied by Sigma-Aldrich (Barcelona, Spain). Phosphatidylcholine (PL) was purchased from Lipoid^®^ (GmbH, Ludwigshafen am Rhein, Germany), and CS was obtained from HMC+ (GmbH, Saale, Germany). Carbomer^®^ 934 (CB) was obtained from Fagron Iberica (Barcelona, Spain). Thyme oil was a sample from Ventós (Barcelona, Spain). Milli-Q water was obtained from a double-distilled Millipore system. All chemicals and reagents used were at an analytical grade.

Clostridium reinforced medium (CRM), Sabouraud Dextrose Agar (SDA), Culture media Brain Heart Infusion (BHI), and Tryptone Soy Agar (TSA) were purchased from Oxoid (Basingstoke, UK). Dulbecco’s Modified Eagle Medium (DMEM) and fetal bovine serum (FBS) were acquired from Thermofisher (Barcelona, Spain). Berens cosmetic diluent was supplied by Scharlab (Barcelona, Spain). MTT and DMSO were purchased from Sigma-Aldrich (Barcelona, Spain).

### 4.2. Preparation of THO-NPs

PLGA NPs were obtained by solvent displacement evaporation using 0.9% PLGA and 0.25% of THO [29]. These parameters were based on previously synthetized NPs-loaded thymol [9,10]. Briefly, an organic phase was formed, made of acetone-solving PLGA and THO, and an aqueous phase was also developed, consisting of either PX or PL at 0.8%. Additionally, for nanoparticles covered with CS, CS was added to the aqueous phase, previously dissolved in 1% acetic acid up to a final concentration of 0.05%. To obtain the NPs, the organic phase was added dropwise into the aqueous phase, under magnetic stirring, and the acetone was removed afterwards using a rotatory evaporator Buchi R-210/215 (Flawil, Switzerland) under a reduced pressure.

### 4.3. Characterization and Stability of THO-NPs

The morphometry of THO-NPs was analyzed using a ZetaSizer Nano ZS (Malvern Instruments, Malvern, UK), measuring Z_av_, PI, and ZP by DLS. To undertake this characterization, samples were diluted in Milli-Q water (1:20). These physicochemical parameters were also assessed to study their stability under storage at 4 °C for 6 months.

### 4.4. In Vitro Antimicrobial Efficacy

The antimicrobial activity of THO and THO-NPs were assayed using the suspension test method, as previously described in our earlier work. Briefly, *C. acnes* was cultured for 48 h in a BHI medium under anaerobic conditions using parches (AnaeroGen^®^, Oxoid, Basingstoke, UK) and indicators (Oxoid, Basingstoke, UK). The experiment was performed, inoculating the samples (either THO or THO-NPs) at a concentration of 250 µg/mL, and incubating them at 37 °C in a shaker incubator (Innova 4080, New Brunswick Scientific, Edison, NJ, USA) for 30 min. Then, 100 µL of each sample was neutralized (1:10) in Beren’s diluent (Scharlab, Barcelona, Spain) for 15 min [30]. Moreover, in order to be used on the drop count method, tenfold dilutions (10 µL) were added to clostridium reinforced medium (CRM) agar dishes. In order to carry out the microbial count, 48 h of incubation under anaerobic conditions at 37 °C was applied. The bacterial viability was expressed as CFU/mL. Statistical analysis was developed by one-way ANOVA (Tukey’s Multiple Comparison Test).

### 4.5. In Vitro Cytotoxicity

The cell viability assay was performed in human keratinocytes cells (HaCaT) by MTT assays via the reduction of tetrazolium salt. Cells were cultured in high glucose DMEM, supplemented with 10% fetal bovine serum (FBS), 100 units/mL penicillin G, 100 µg/mL streptomycin 2 mM, and L-glutamine, and was then incubated at 37 °C (5% CO_2_) until cells reach a confluence of 80–90%. Cells were seeded as described elsewhere, and THO-NPs were used at different concentrations (2, 10, and 20 µg/mL), then diluted in DMEM. After the incubation for 24 h at 37 °C (5% CO_2_), the medium was removed, MTT was added (0.25% in PBS), and, after 2 h, the medium was replaced by 100 µL DMSO. Finally, cell viability was then determined as 570 nm in a Modulus^®^ Microplate Photometer (Turner BioSystems Inc., Sunnyvale, CA, USA). Results were calculated as the percentage of living cells against untreated ones.

### 4.6. In Vitro Antioxidant Activity

The antioxidant activity of THO-NPs was studied in HaCaT cells by measuring reactive oxygen species (ROS) [31]. ROS were measured using the fluorogenic probe H_2_DCFDA (2′,7′-dichlorodihydrofluorescein diacetate). To carry out this measurement, 100 µL of cells were seeded for 72 h in 96-well plates (2 × 10^5^ cells/well), and stained for 45 min in the dark with the fluorogenic probe, diluted in DMEM (at 25 µM), without FBS and phenol red. Afterwards, cells were washed and incubated with either THO, THO-NPs, or blank NPs for 2 h. After that, oxidation processes were induced, adding 10 µL in each well of H_2_O_2_ 20 mM. Cells with or without H_2_O_2_ were used as positive and negative controls, respectively. To undertake the measurement, cells were measured with a plate fluorometer (Varioskan, Thermo Fisher Scientific, Rockford, IL, USA) at λ_ex_ 485 nm and λ_em_ 530 nm. Data were acquired from the initial addition time of up to 120 min, and the positive control (H_2_O_2_) at the last measure was extrapolated as 100% oxidation. The fluorescence background (negative control) was subtracted from the measurements. Data were analyzed and processed using GraphPad^®^ Prism 6 software (Version 6) and statistical analysis was developed either by *t*-test or by one-way ANOVA (Tukey’s Multiple Comparison Test).

### 4.7. In Vitro Anti-Inflammatory Efficacy

This experiment was performed as previously described in our earlier work [10]. Briefly, *C. acnes* fresh inoculum was cultured under anaerobiosis in BHI for 5 days (growth until the stationary phase), to be used as inflammatory inducer. Moreover, HaCaT cells were adjusted (2 × 10^5^ cells/well), seeded in 12-well plates and incubated for 48 h (37 °C, 5% CO_2_). After this time, they were treated with THO, THO-NPs, or NPs (blank NPs) during 2 h. After this time, cells were stimulated for 4 h with *C. acnes*. Cells with and without *C. acnes* incubation were used as negative and positive controls. Total RNA was isolated from cells using the extraction kit (Qiagen RNeasy, Germantown, MD, USA) and quantified, using a NanoDrop TM-2000 spectrophotometer at 260 and 280 nm, by the absorbance ratio. The cDNA was synthesized from RNA using High-Capacity cDNA Reverse Transcription kit (Applied Biosystems, Foster City, CA, USA). Quantitative PCR reactions were performed using SYBR^®^ Green PCR Master Mix and specific human oligonucleotide primers for IL-8, IL-6, TNF-α, IL-1β, and β-actin (endogenous control), using a StepOne Plus PCR cycler (Applied Biosystems, Foster City, CA, USA). PCR program was conducted by denaturation cycle (10 min, 95 °C), 40 cycles of 15 s at 95 °C and 1 min at 60 °C. Relative gene expression was calculated as fold-change compared to sample control by means of 2−ΔΔCt formula. Data were analyzed and processed using GraphPad^®^ Prism 6 software and statistical analysis was developed by one-way ANOVA, followed by Tukey’s Multiple Comparison Test.

### 4.8. Wound-Healing Activity

Wound-healing activity was studied in HaCaT cells seeded in 12-well plates at 5 × 10^4^ cells/well density and grown until 70–80% confluence. After this, each well was cross scratched in the centre, using a 200 µL pipette tip, washed with PBS, and refilled with DMEM containing 1% FBS. To evaluate the wound-healing activity, cells were treated for 2 h with THO, THO-NPs, or their corresponding empty NPs, and then afterwards washed with PBS and incubated for 24 h in 1% FBS DMEM [27,32]. Images were recorded by means of a fluorescent microscope at 10× (LEICA DFC300FX) before the treatment and after 24 h incubation. They were processed with ImageJ software (v1.53t).

### 4.9. Ex Vivo Skin Antioxidant Efficacy

The antioxidant activity was measured using ex vivo pig skin and methylene blue [33]. Ex vivo pig skin was obtained from the animal house (Bellvitge, University of Barcelona, Barcelona, Spain), and was used in accordance with the protocol approved by the Ethics Committee of the University of Barcelona. Therefore, skin samples were cut into 2 cm^2^ and put with the SC facing up into a 6-well plate, containing PBS (0.5 mL) to keep the bottom of the tissue moisture. Then, 50 µL of methylene blue 0.01% was added onto the skin surface and incubated at 32 °C for 4 h. Skin samples were PBS-washed and dried using filter paper on the SC. After this procedure, either samples (30 µL) or distilled water (as the control) were applied on the skin, and incubated again during 1 h. Images were recorded right after administering the formulations and after 1 h of the treatment at 32 °C [10].

### 4.10. Preparation of THO Hydrogels

For the preparation of THO-NPs hydrogels, the aqueous suspension was incorporated into the total water content of the final formulation, containing 5% GLY and 5% PG. For the THO hydrogel, this was previously dissolved in these solvents using a sonication bath, then incorporated into the water. In both cases, the final concentration of THO was 0.1%. Additionally, 0.5% CB was added to the formulations and mixed using Unguator^®^ (Microcaya, Bilbao, Spain). Then, the pH was adjusted to 5.0–5.5 with NaOH 2N, under vigorous continuous stirring until the viscous hydrogel was formed.

### 4.11. Characterization and Stability of the Hydrogels

The morphometry of GC-THO-NP-L- was analyzed by DLS measuring Z_av_ and PI and followed stability under storage at RT for 6 months. Therefore, dilutions were performed in Milli-Q water (1:200). Gel matrix and its homogeneity were assessed by means of an optical microscopy X63 (LEICA DFC300FX, Leica Microsystems, Wetzlar, Germany) was used.

The hydrogel stability was studied FOR up to 6 months at room temperature (RT, 25 ± 2 °C), and up to 12 months at 4 °C, observing the appearance, color, odor, and pH. Measurements of Z_av_ and PI were recorded along storage for 6 months at 4 ± 2 °C.

Additionally, the stability of hydrogels was assessed through measuring the BS profile. This was performed to evaluate the short-term stability behavior of the NPs within the gel matrix. Scans were run for 24 h every hour, as well during storage at RT for 6 months.

The microbial preservative sterility of developed formulations was evaluated after 6 months of storage at RT. For this, 0.1 g of hydrogel or 1 mL of the aqueous suspension was transferred into TSA and SDA Petri dishes, for bacteria and yeast growth media, respectively. Microbial absence was evaluated after incubation (35 ± 2 °C for 3 days or at 28 ± 2 °C for 7 days, for bacteria and yeast, respectively). This methodology was developed following the specifications stated on the European Pharmacopeia monographs (2.6.12. Microbiological examination of non-sterile products: total viable aerobic count), as previously described in our earlier work [9].

### 4.12. Rheology Studies

The rheological properties of the THO-NPs hydrogels were determined with a cone-and-plate geometry rotor (Haake C60/2Ti, 6 cm diameter) using rheometer Haake Rheo Stress 1 (Thermo Fisher scientific, Kalsruhe, Germany) [34,35]. For rotational testing, the shear stress (τ) and viscosity (η) at 25 ± 0.1 °C were evaluated continuously to measure the shear stress (Pa) as a function of the shear rate γ (s^−1^). The shear rate ramp was set up at a constant rate of 100 s^−1^ for 1 min, including the ramp-up period from 0 to 100 s^−1^ (3 min), and the ramp-down period. Data measurements were analyzed using Haake RheoWin^®^Data Manager V 3.3 software. The flow curves were fitted using mathematical models. Data adequacy to the models was based on the correlation coefficient value (r) and the chi-square value. Furthermore, the viscosity (η, Pas) was determined from the constant shear section at 100 s^−1^.

### 4.13. Extensibility Studies

The extensibility of GC-THO and GC-THO-NP-L- was studied under compression, by applying increasing weights at room temperature. For this, hydrogel samples (0.5 g) were placed onto the gap holder and covered with the top-slide (26.06 g). Thus, the applied weights were held for 60 s consecutively, and each measure (cm) of the diameter (circle) obtained was recorded. The extensibility of the hydrogels was then determined by the increased surface area (cm^2^), calculated by Equation (1) as the function of the increasing weights [36,37]:

(1) $$E=\frac{\pi .{\left(d\right)}^{2}}{4}$$

where *d* is the average diameter measured in different directions.

### 4.14. In Vivo Biomechanical Properties on Skin

The skin integrity was evaluated through the hydration levels of the SC (Corneometer^®^) and by the TEWL^®^ in the forearm of 10 healthy volunteers for each product. The study was approved under the code IRB00003099 by the Ethics Committee of UB (30 January 2019). The biomechanical properties of the semisolid formulations were determined through measuring the TEWL (DermaLab^®^ module, Cortex Technology, Hadsund, Denmark) and the skin hydration of the SC (Corneometer^®^ CM 825, C+K electronic, Courage-Khazaka electronic GmbH, Cologne, Germany). Measurements were taken before and after the application of the GC-THO and GC-THO-NP-L- formulations, performed in a climate-controlled room (25 ± 2 °C and 45% relative humidity) at selected timepoints, including a prior adaptation period (30 min). The statistical data were processed by GraphPad^®^ using ANOVA non-parametric system, Wilcoxon paired test [37].

## Figures and Tables

**Table 1:** Physicochemical parameters and their stability for 6 months when stored at 4 °C for the developed surface-functionalized THO-NPs (Surface functionalization: PX 0.8%, PL 0.8% or C 0.05%).

	t (Months)	Z_av_ (nm)	PI	Z_P_ (mV)
THO-NP-L-	0	193.3 ± 1.1	0.153 ± 0.029	−32.1 ± 0.9
	3	228.9 ± 19.6	0.154 ± 0.061	−24.3 ± 0.8
	6	254.8 ± 3.6	0.173 ± 0.018	−18.1 ± 0.5
THO-NP-P-	0	181.2 ± 4.9	0.052 ± 0.014	−22.8 ± 0.2
	3	193.2 ± 3.1	0.123 ± 0.032	−17.1 ± 0.3
	6	196.0 ± 4.1	0.123 ± 0.056	−13.9 ± 0.6
THO-NP-P-C+	0	237.5 ± 2.9	0.168 ± 0.028	24.3 ± 0.7
	3	326.9 ± 9.6	0.167 ± 0.06	20.1 ± 0.2
	6	344.4 ± 8.3	0.174 ± 0.02	16.5 ± 0.4

**Table 2:** The physicochemical parameters and stability of GC-THO-NP-L-.

	t (Months)	Z_av_ (nm)	PI
GC-THO-NP-L-	0	268.0 ± 6.5	0.306 ± 0.080
	3	293.6 ± 16.5	0.374 ± 0.112
	6	297.9 ± 38.4	0.364 ± 0.109

**Table 3:** Rheology and extensibility parameters for the developed hydrogels.

	Parameters	GC-THO	GC-THO-NP-L-
Rheology	Viscosity at 100 s^−1^ (Pa·s)	1.01 ± 0.00	0.37 ± 0.00
	Flow	Shear thinning	Shear thinning
$\eta =\frac{\tau 0}{y+K*y^{\left(m-1\right)}}$	Equation	Herschel–Bulkley	Herschel–Bulkley
	r (up/down)	1/1	1/1
Extensibility	Eₘₐₓ	25.09 ± 2.21	45.71 ± 4.12
	K_m_	37.02 ± 3.81	31.44 ± 3.91
$E=\frac{E_{\max}\cdot w}{K+w}$	Equation	Hyperbola	Hyperbola
	r^2^	0.9347	0.9611

*η*: apparent viscosity, *τ*_0_: shear stress (Pa) when deformation velocity tends to zero, *y*: deformation velocity, *K* consistency coefficient, *m:* flow index, *E*: extensibility, *E*_max_: maximum extensibility value; *K_m_*: weight necessary to reach half of the maximum extensibility, *w*: weight.

## Data Availability

The data presented in this study are openly available in article.
